# Immunotherapy of Cancer: Reprogramming Tumor-Immune Crosstalk

**DOI:** 10.1155/2012/760965

**Published:** 2012-10-11

**Authors:** Kyle K. Payne, Amir A. Toor, Xiang-Yang Wang, Masoud H. Manjili

**Affiliations:** ^1^Department of Microbiology & Immunology, Massey Cancer Center, Virginia Commonwealth University, Richmond, VA 23298, USA; ^2^Department of Internal Medicine, Massey Cancer Center, Virginia Commonwealth University, Richmond, VA 23298, USA; ^3^Department of Human and Molecular Genetics, Massey Cancer Center, Virginia Commonwealth University, Richmond, VA 23298, USA

## Abstract

The advancement of cancer immunotherapy faces barriers which limit its efficacy. These include weak immunogenicity of the tumor, as well as immunosuppressive mechanisms which prevent effective antitumor immune responses. Recent studies suggest that aberrant expression of cancer testis antigens (CTAs) can generate robust antitumor immune responses, which implicates CTAs as potential targets for immunotherapy. However, the heterogeneity of tumor cells in the presence and quantity of CTA expression results in tumor escape from CTA-specific immune responses. Thus, the ability to modulate the tumor cell epigenome to homogenously induce expression of such antigens will likely render the tumor more immunogenic. Additionally, emerging studies suggest that suppression of antitumor immune responses may be overcome by reprogramming innate and adaptive immune cells. Therefore, this paper discusses recent studies which address barriers to successful cancer immunotherapy and proposes a strategy of modulation of tumor-immune cell crosstalk to improve responses in carcinoma patients.

## 1. Introduction

Conventional approaches in the therapy of cancer, such as chemotherapy, have shown only modest success in the treatment of advanced carcinoma [1]. Historical comparisons since the late 1970s have shown that the introduction of combination cytotoxic chemotherapy has produced a modest 9–12 month gain in survival compared with untreated breast cancer patients [2]. Despite advances in conventional cytotoxic therapies of early-stage breast cancer [3, 4] there remains no therapeutic strategy that can ensure relapse-free survival. Furthermore, studies have shown that 20% of clinically disease-free early-stage breast cancer patients relapse within 10 years after conventional therapies [5]; indeed, most cancer-related deaths within the United States are attributed to relapse [6]. Thus, there is an urgent need to develop more effective therapies to overcome breast cancer relapse and to treat advanced cancer. To this end, immunotherapy emerges as promising strategy for the prevention of tumor relapse, when combined with conventional therapies. 

Thus far advances in the immunotherapy of cancer have also been met with a number of setbacks. Several vaccination strategies used against breast cancer have been successfully employed to induce tumor-specific CD8^+^ and CD4^+^ T-cell responses; however, such immunological responses have rarely been potent enough to achieve objective results [7–9]. Additionally, it has been demonstrated by several groups that adoptive cellular therapy (ACT) directed against highly immunogenic melanoma-associated antigens results in objective responses in animal models as well as in some melanoma patients [10, 11]. ACT has also been tested against breast cancer both in preclinical and clinical studies [12, 13]; however, unlike melanoma, ACT has not produced promising results in breast cancer patients and has only displayed effectiveness in animal models in prophylactic settings [14, 15], rather than against well-established, vascularized tumors. Such failure has been attributed, in part, to (i) the lack of a robust antitumor immune response as a result of the expression of weakly immunogenic tumor antigens coupled with the presence of low frequency and low affinity T cells and (ii) the suppression of antitumor immune responses though the activity of immunosuppressive mechanisms. Indeed, distant recurrence of breast cancer may occur even in the presence of tumor-specific immune responses. The ability to overcome these barriers will likely improve the efficacy of immunotherapy directed against cancer. To address these issues, the crosstalk between tumor cells and cells of the immune system should be altered in order for reprogrammed tumor cells and immune cells to prevent tumor relapse as well as induce regression of advanced cancer. 

## 2. Immune Suppression 

It is now well established that the mammalian immune response can be suppressed through various mechanisms. The expression of immunoregulatory molecules, such as CTLA-4 and PD-1 as well as the ectoenzyme, CD73, inhibits the proliferation and function of conventional T cells [16, 17]. Furthermore, immunosuppressive cells such as alternatively activated M2 macrophages, type II NK cells, and regulatory T cells have been demonstrated to antagonize tumor immunosurveillance [18–22]. 

Results from clinical studies involving breast cancer patients indicate that another critical regulator of tumor immunosurveillance, the myeloid-derived suppressor cell (MDSC), was found to be the most abundant type of suppressor cell [23, 24] and thus represent a major hurdle in overcoming antitumor immune suppression. MDSCs represent a phenotypically heterogeneous population of myeloid cells at different stages of maturation. These cells have been found in tumor-bearing mice as well as cancer patients and have been shown to possess multiple mechanisms to suppress the antitumor immune response [25, 26]. Such responses include disrupting TCR antigen recognition and T-cell mediated IFN-*γ* production [27, 28], depletion of essential amino acids within the tumor microenvironment [29], and overproduction of reactive oxygen species (ROS) [30]. Murine MDSCs are defined as coexpressing Gr-1 and CD11b, with two subsets commonly being described: granulocytic (CD11b^+^Ly-6G^+^Ly-6C^low^) and monocytic (CD11b^+^Ly-6G^−^Ly-6C^high^) [31]. Human MDSCs, on the other hand, have been difficult to be identified as initial studies revealed that these cells express varied phenotypes and suppressive patterns [25]. It is now regarded, however, that human MDSCs fall into two main subsets: a monocytic population characterized by expression of CD14 and a granulocytic population characterized by CD15 expression; both subtypes have been reported to express the common myeloid markers CD11b and CD33, with minimal expression of myeloid maturation markers such as HLA-DR [32]. The accumulation of these cells in association with cancer development is corroborated by experimental mouse models, indicating that MDSCs develop as a function of tumor progression [33]. For instance, our group has previously reported that FVBN202 mice, which overexpress the rat neu oncogene in their mammary glands, develop atypical ductal hyperplasia (ADH) and ductal carcinoma in situ (DCIS) in mammary epithelial cells prior to the formation of spontaneous mammary tumors [34]. DCIS of the breast is conventionally regarded as a precursor of invasive breast cancer, and ADH is a risk factor for the development of the disease [35, 36]. Compromised antineu immune responses occur as a result of the emergence of premalignant events, such as ADH and DCIS, which are characterized by an accumulation of MDSCs in the blood, bone marrow, secondary lymphoid tissues, and within tumor lesions due to an increased production of tumor-derived soluble factors [34, 37–41]. Such findings provide evidence that MDSCs function as potent inhibitors of antitumor immunity in breast cancer models. Likewise, human MDSCs have been observed to negatively regulate both adaptive and innate immunity during cancer development and progression, with accumulation having been observed in peripheral blood and lymphoid tissues as well as draining tumor sites of cancer-bearing patients [31]. In addition to breast cancer, the accumulation of MDSCs has been observed in other neoplastic diseases, such as hepatocellular, pancreatic, esophageal, and colorectal cancers [26], and is generally correlated with advanced clinical cancer stage and metastatic tumor burden with a demonstrated suppression of antitumor immune responses correlating with poor responses following conventional therapies [14, 23, 42, 43]. Thus, MDSC accumulation is paramount in the ability of cancer to evade effective immune responses. Therefore, suppression of immune responses mediated by MDSCs must be overcome to rescue and facilitate effective tumor-specific immunity. Accordingly, it was reported that activated NKT cells can overcome MDSCs, thereby supporting an effective adaptive immune response against cancer [15, 44, 45]. Our group has recently developed a novel strategy of reprogramming immune cells ex vivo to overcome MDSC-mediated antitumor immune suppression in a prophylactic model of murine breast carcinoma upon adoptive transfer, which resulted in a demonstrated ability to enhance immune mediated rejection of tumors [15]. However, this approach failed to protect mice in a therapeutic model against established tumors. Thus, in addition to overcoming MDSC-mediated suppression, improvements in the efficacy of immunotherapy likely will require further addressing the crosstalk between immune and tumor cells; one such strategy is enhancing tumor cell immunogenicity. 

## 3. In Situ Vaccination: Modulating the Tumor ****Cell Epigenome

A barrier for successful immunotherapy of breast cancer is the low immunogenicity of tumor cells, for example, expression of tumor associated antigens which are recognized as “self” by the immune system. Therefore, improving the immunogenicity of tumor is essential to improving tumor immunotherapy. To this end, in situ induction of foreign-like antigens, such as cancer testis antigens (CTA), to which T-cell tolerance does not exist, is a promising option. CTAs are highly immunogenic with no natural self-tolerance due to the observation that they are normally only expressed during embryonic development; after birth, expression is generally limited to immunologically privileged germ cells and the placenta [46]. Aberrant CTA expression was first described in melanoma; as such, this expression was found to generate CTA-specific cytotoxic T-cell responses [47]. Recently, it was reported that treatment of metastatic melanoma with autologous CD4^+^ T cells specific for the CTA, NY-ESO-1, elicited long-term complete remission [48]. In addition to melanoma, CTA expression has also been observed in hematological malignancies [49] as well as solid tumors, including breast cancer [50, 51]. Further, CTA expression in breast cancer has been shown to elicit a broad range of cellular and humoral immune responses [50, 52, 53]; both CD8^+^ T cell and CD79^+^ B cell infiltration has been observed in primary and metastatic NY-ESO-1 expressing breast cancer [54]. Of note, a significantly elevated expression of NY-ESO-1 and MAGE-A, another highly immunogenic CTA, was detected in triple negative breast cancers compared to other types of breast cancer [55], which therefore represent antigenic targets in an otherwise immunologically refractory breast cancer subtype.

Importantly, CTA expression is normally silenced by methylation within the promoter region of these genes. Methylation at the C-5 position of cytosine bases within DNA is a covalent chemical modification which characterizes a key, biologically functional, epigenetic modification of the animal genome [56]. This action primarily occurs at CpG dinucleotides in mammals, where DNA-methyltransferases (DNMTs) mediate the transfer of methyl groups to cytosine, thereby generating 5-methylcytosine (5mC) that has been shown to play a critical role in the cellular protein expression by transcriptional silencing of genes [57]. Aberrant CTA expression likely occurs due to epigenetic molecular alterations which arise during tumor progression; cancer cells display drastic changes in DNA methylation status, typically exhibiting global DNA hypomethylation as well as region-specific hypermethylation [58], resulting in irregular expression of CTAs. Our group has observed that a lack of such aberrant CTA expression within breast tumor lesions at the time of diagnosis correlated with eventual relapse after conventional therapies (unpublished data) along with the lack of expression of an immune function gene signature [59]. Conversely, the tumors in patients who remained free of relapse expressed both CTAs and the immune function gene signature. These data suggest that CTA expression in breast cancer patients activates effective immune responses which results in improved prognosis after conventional treatments.

In order to induce and/or increase expression of CTAs to function as target antigens and improve the prognosis in patients with breast cancer, it is possible to modulate the tumor epigenome to initiate the cellular CTA transcriptional program; such an approach will serve to impart a more immunogenic tumor cell phenotype. Azacitidine (Aza) and Decitabine (Dec) are both hypomethylating agents employed in epigenetic therapy to modify cellular methylation patterns; both of these agents have been approved for clinical use in the treatment of myelodysplastic syndrome. Aza and Dec function as cytosine analogs, which lead to their incorporation into newly synthesized DNA strands during S phase of the cell cycle; these agents have been shown to induce and/or increase the expression of various CTAs in a variety of in vitro and in vivo tumor models [49, 52–54]. Both Aza and Dec have demonstrated the ability to induce the expression of CTAs, as well as the tumor suppressor gene p53 [60] and the death receptor Fas [61] on tumor cells. These are attributed to their capacity to function as potent DNMT inhibitors through the formation of a covalent complex with a serine residue at the active site of DNMT1, which therefore results in CpG island demethylation during cellular proliferation. This, in turn, results in hypomethylation within the promoter of tumor suppressor genes as well as a highly immunogenic CTAs [56, 62–64], thereby rendering tumor cells susceptible to CTA-reactive immune responses and suppression of proliferation via expression of p53, as well as rendering these tumor cells more susceptible to Fas L-induced apoptosis by CTA-reactive T cells. Such modulation of CTA expression using Aza has been shown to generate CTA-specific T-cell responses in patients with acute myeloid leukemia, as demonstrated by our group [65]. Others have demonstrated the feasibility to induce CTA expression in vivo using Dec in the 4T1 model of murine breast carcinoma, resulting in greater tumor cell cytotoxicity upon treatment with CTA-specific T cells [56]. Further, an ongoing clinical trial in breast cancer patients is testing the efficacy of Dec for the induction of the expression of ER/PR in patients with hormone receptor negative tumors in order to render them susceptible to hormonal therapy [66]. 

Decitabine is a particularly attractive option to induce CTA expression as it functions as a prodrug which requires activation by deoxycytidine kinase (DCK), an enzyme preferentially expressed in tumor cells and myeloid cells. Thus, the effects of Dec are likely tissue specific, as DCK is selectively expressed in tumor cells and myeloid cells, thus protecting T and B cells from the potentially deleterious demethylating effects of this agent. In addition, DCK has been found to be overexpressed in poor outcome breast cancer [67], suggesting that epigenetic therapy to induce CTA expression may prove to be an efficacious approach in breast cancer patients with poor prognosis. 

Our group has recently demonstrated that epigenetic modulation using sequential Aza and the immunomodulatory agent lenalidomide for the induction of CTA expression in the tumor and CTA-specific antitumor immune responses in patients with multiple myeloma [65]. Upon determination of CTA expression in bone marrow of multiple myeloma patients following treatment with Aza, we found that CTA expression is induced exclusively in CD138^+^ malignant plasma cells in vivo, which suggests a preferential induction of hypomethylation in CTA promoters within tumor cells. As a result of such a strategy, which we term in situ vaccination or epigenetic induction of an adaptive immune response, we have determined that the observed induction of CTA expression resulted in the generation of robust CTA-specific adaptive immune responses [65]. We believe that this strategy will maintain long-term surveillance against malignant plasma cells in patients with MM and translate into prolonged freedom from progression in this otherwise incurable disease. Furthermore, these data suggest that epigenetic therapeutic agents, such as Dec, when used in a neoadjuvant setting, may induce CTA expression in tumor-bearing patients and may therefore activate early CTA-specific immune responses to prevent recurrence after conventional therapies.

## 4. Reprogramming of Tumor-Sensitized Immune Cells

The rationale for ex vivo reprogramming of tumor-sensitized immune cells is based on overcoming the low frequency of endogenous tumor-reactive T cells by driving their expansion and activation toward the most effective antitumor phenotype(s). We have previously shown the ability of ex vivo reprogrammed Her2/neu sensitized immune cells to protect mice in a prophylactic setting when used in an adoptive cellular therapy (ACT) setting [15]. Cellular reprogramming through the combined use of bryostatin 1, a potent activator of classical and novel protein kinase C (PKC) [68, 69], and ionomycin (B/I), a calcium ionophore [70, 71], followed by differentiation using gamma-chain (*γ*-c) cytokines (IL-2, IL-7, and IL-15) results in the ability to selectively activate tumor-primed T cells, NK cells, and NKT cells, as described by our group [72, 73]. In particular, the generation of both CD4^+^ and CD8^+^ central memory (CD44^+^ CD62L^high^) lymphocytes, which are necessary to mediate protection in ACT recipients upon challenge with antigen expressing tumor cells, is observed. Furthermore, we observed that reprogrammed NK/NKT cells surprisingly functioned to render T cells resistant to MDSC suppression and induced tumor rejection even in the presence of MDSC in FVBN202 mice [15]. 

Therefore, it may prove beneficial to harvest autologous peripheral blood mononuclear cells (PBMC) from breast cancer patients having received neoadjuvant Dec treatment in order to reprogram CTA-sensitized immune cells using B/I and *γ*-c cytokines; following conventional therapies, such reprogrammed lymphocytes can then be reinfused back into the host, whereupon they may exert long-lived protection against relapse, even in the presence of classical immunosuppressive cells such as MDSCs. 

## 5. Rescue of Late Antitumor Immune Responses

We have previously demonstrated that MDSC accumulation results as a function of tumor-derived soluble factors, such as GM-CSF, in the FVBN202 model of breast carcinoma [74], while others have identified additional tumor-derived soluble factors and inflammatory cytokines which are responsible for the accumulation of MDSCs [38–41]. We have also verified the ability of radiation therapy (RT) to reject primary tumors, thereby resulting in the reduction of MDSCs within the tumor bearing host [15]. Ionizing irradiation is known to cause cellular stress and enhance the synthesis of a variety of immune-stimulatory and -modulating molecules such as heat shock proteins (HSP) [75, 76], high mobility group box 1 (HMGB1) [76], and NKG2D ligands [77]. Such danger signals are then sensed by cells of the immune system. For instance, toll-like receptor (TLR)-4 on DCs interacts with its ligands including HMGB1 [78] and HSPs [79] and enhances maturation and antigen presentation capacity of DCs. Detection of danger signals in tissues by leukocytes activates an immune response involving cells of the innate (myeloid and NK cells) and adaptive (T and B cell) lineages. RT-induced NKG2D ligand, an activating receptor for NK cells, and HSP70 render tumor cells more susceptible to NK-cell-mediated cytolysis [80]. Thus, combining RT with an enhanced immunotherapeutic strategy, such as neoadjuvant administration of Dec, is likely to enhance antitumor immune responses and produce objective responses against advanced breast cancer and result in a decreased risk of disease relapse. The removal of MDSC-mediated suppression via RT may, therefore, facilitate the rescue of CTA-specific antitumor immune responses against residual tumor cells and result in the prevention of future disease recurrence. Accordingly, we propose that CTA-reactive T cells became antigen experienced during tumorigenesis due to aberrant CTA expression; however, it is likely that such CTA expression occurs late in the progression of the tumor, thus rendering CTA-reactive T cells ineffective due to MDSC accumulation via tumor-derived soluble factors. It is expected, nevertheless, that patients who receive neoadjuvant Dec followed by radiation therapy or surgery to remove the primary tumor will experience a reduction in MDSC accumulation; we propose that such activity will result in the rescue of CTA-reactive T cells from suppression to eliminate residual tumor cells in order to decrease the likelihood of future disease recurrence. 

## 6. Limitations and Future Considerations

The majority of solid tumors and hematological malignancies undergo a period of dormancy that is characterized by years to decades of minimal residual disease (MRD) in which cancer progression has paused [81, 82]. Indeed, disease-free periods in breast cancer patients can last as long as 25 years and are clearly associated with the presence of MRD; subsequent relapse represents the escape of the tumor from dormancy, which can include locoregional recurrence as well as distant metastatic disease [81, 83, 84]. Tumor dormancy may be the result of hypoxic stress, as well as other as yet unknown cues from the microenvironment of the host [85]. The mechanism of tumor cell dormancy may best be explained by cellular quiescence. Quiescence is defined as growth/proliferation arrest and is thought to be due to G0-G1 cell cycle arrest, during which cells pause cellular activities which can render them refractory to differentiation and proliferation [86, 87]. Thus, given that DNMT inhibitors Dec and Aza are incorporated into cellular DNA during S phase, the induction of CTA expression requires tumor cells to be actively proliferating. As such, the in situ vaccination strategy outlined above will likely be less effective against any residual tumor cells that have entered G0-G1 arrest. Therefore, further understanding the process by which residual tumor cells naturally exit dormancy may provide novel approaches to coax such cells to exit cell-cycle arrest. Future studies investigating the ability of Aza or Dec combined with histone deacetylase inhibitors (HDI) to reinitiate the cell cycle would be beneficial in addressing this problem. Such efforts may result in an enhanced ability of in situ vaccination strategy to target and eliminate MRD, which may therefore lower the incidence of tumor recurrence presently observed in breast cancer patients. 
